# Different dose of heparin in preventing radial artery occlusion after transradial coronary angiography: A protocol for systematic review and meta-analysis of randomized controlled trials

**DOI:** 10.1097/MD.0000000000023227

**Published:** 2020-11-13

**Authors:** Ling Zhao, Yanlei Pang, Huijing Zhang, Yong Li, Qun Zheng, Fengde Li

**Affiliations:** a Department of Cardiology, the Fourth People's Hospital of Hengshui, NO.485 Xinhua Road; b Department of Cardiology, Hengshui People's Hospital, NO.180 Renmin Road, Taocheng District, Hengshui, Hebei, China.

**Keywords:** anticoagulant therapy, coronary heart disease, heparin, meta-analysis, radial artery occlusion, transradial coronary angiography, transradial interventional therapy

## Abstract

**Background:**

When atherosclerosis occurs in the coronary artery, resulting in stenosis, occlusion, or spasm of the coronary artery, the supply of blood and oxygen to the myocardium will be reduced or even unavailable, resulting in myocardial necrosis and heart pain, chest tightness, dyspnea and other symptoms caused by myocardial necrosis are collectively referred to as coronary atherosclerotic heart disease. Coronary angiography can not only understand the degree of coronary artery damage, but also estimate the prognosis of coronary artery stenting, which provides a reliable reference for clinical treatment. Transradial coronary angiography (TCA) has the advantages of high success rate, small trauma, less complications, no bed rest, reduce hospital stay and other superiority, which accepted and used by physicians. Although the success rate of surgery is high, the postoperative complications will still affect the effect of surgery and the prognosis of patients. The main manifestations are radial artery occlusion (RAO), forearm hematoma formation, pseudoaneurysm formation, periosteal compartment syndrome, radial artery perforation, etc. Among the many ways to prevent RAO, anticoagulant therapy with common heparin is one of them, but the dosage of heparin is not clear. Therefore, we decided to use systematic evaluation to evaluate the clinical effectiveness and safety of different dose of heparin in preventing of RAO, and to provide clinical basis for the early prevention and treatment of RAO.

**Methods:**

Two reviewers independently searched PubMed, Embase, the Cochrance Library, Web of Science, Medline, CBM Disc, CNKI, and WANFANG Data to find the eligible research. The retrieval about the randomized controlled trials of different dose of heparin in preventing the occurrence of RAO after TCA in recent years. The retrieval time is set between January 1990 and June 2020. The retrieval language is Chinese/English. Two researchers independently searched, managed and screened the literature through the search terms. When the 2 parties have inconsistent opinions on the inclusion or not of certain literature, the literature will be referred to the third researcher for discussion and decision. The included studies are conducted bias risk assessment through bias risk assessment tool, which based on Cochrane Handbook 5.0. The extracted data uses RevMan5.3 software for statistical processing.

**Results:**

The research results of this systematic review will be published in peer-reviewed medical-related academic journals.

**Conclusion:**

This study adopts the Meta-analysis method and expands the sample size, which will give high-quality evidence-based medicine evidence on the clinical effectiveness and safety of different dose of heparin in preventing the occurrence of RAO.

**Trial registration number:**

OSF, DOI 10.17605/OSF.IO/CPXJ3.

## 1 Introduction

Coronary heart disease is abbreviated as coronary heart disease, it is one of the most common cardiovascular diseases in clinical practice.^[1,2]^ The pathogenesis of coronary heart disease is characterized by myocardial ischemia and hypoxia caused by insufficient blood supply of coronary artery, which leads to chest tightness, chest pain, shortness of breath, palpitations and other uncomfortable symptoms. In severe cases, related complications can occur: malignant arrhythmia, refractory heart failure, cardiogenic shock, cardiac rupture, etc. Coronary heart disease has become a major public health problem facing human beings worldwide.^[3–5]^ Epidemiological surveys have shown that the global prevalence of cardiovascular diseases has continued to increase in recent years. The medical burden of cardiovascular disease is increasing day by day. As one of the cardiovascular diseases, the incidence and mortality of coronary heart disease are also increasing year by year. Therefore, early detection, early diagnosis and timely treatment of patients with coronary heart disease are very important to improve the survival rate of patients with coronary heart disease and improve the long-term prognosis of patients.^[6,7]^

Coronary angiography is currently the gold standard for the diagnosis of coronary heart disease, and percutaneous coronary intervention is a safe, reliable and invasive method for the treatment of coronary heart disease in cardiovascular medicine. According to the degree of individualization of patients, most of these 2 methods of operationare most widely used through radial artery, brachial artery and femoral artery.^[8,9]^ Comparing with the brachial artery and femoral artery routes, transradial coronary angiography (TCA) has the advantages of less damage to the body, faster recovery, shorter hospital stays, no need for bed rest, and reduces postoperative complications. With its unique advantages, its effectiveness has been widely recognized by cath lab surgeons in recent years, and it has become one of the most acceptable surgical approaches for patients.^[10]^ Despite its unique advantages in safety, the complications of TCA still exist, which are mainly manifested as radial artery occlusion, forearm hematoma formation, pseudoaneurysm formation, compartment syndrome, radial artery perforation, etc.^[11]^ The most common clinical complication is radial artery occlusion (RAO). Statistically, the probability of RAO after TCA is 6% to 10%. Due to the small diameter of the radial artery, once RAO is caused after TCA, although the symptoms and function of hand will not be damaged due to the dual blood supply of palm, it may cause adverse mental impact on patients, and also will inevitably affect the selection of radial artery approach in patients with coronary intervention again. Therefore, exploring the risk factors of RAO and avoid or reduce RAO, which has the practical and clinical significance.^[12]^

There are many ways to prevent RAO, the more conventional methods include using gentle and skilled techniques to reduce the damage to the radial artery during the preoperative puncture process, rational use of anticoagulant drugs, the use of non-obstructive hemostasis, the reduction of compression hemostasis time, preoperative injection of nitroglycerin, reasonable pain relief and so on.^[13]^ Among them, the rational use of anticoagulant drugs in surgery is one of the effective methods to prevent RAO, and it is widely used in operation at present. Common heparin as the most classic anticoagulant, it is still the first choice of anticoagulant in surgery. As far as simple coronary angiography is concerned, whether the dose of heparin used during the operation should be calculated according to the patients weight and added to complete heparinization in batches has not been clearly determined.^[14]^ Most of the patients who underwent coronary angiography used heparin of 2000IU-5000IU to prevent the complication of RAO. According to the difference of different countries, doctors habits and patients individualization, the dose of heparin used in coronary angiography is usually conventional dose (2000IU-3000IU or 50IU/kg) and high-dose (5000IU or 100IU/kg). However, there are few literatures comparing the clinical effects of these 2 methods of using heparin. Therefore, whether to use high-dose heparin is better than low-dose heparin in preventing of postoperative RAO, and whether the use of high-dose heparin be used in transradial coronary angiography to effectively prevent the occurrence of RAO without increasing the risk of bleeding and other related issues are still controversial.^[15,16]^ This study will search recent randomized controlled trials of different dose of heparin in preventing of RAO after TCA. Meta-analysis was used to expand the sample size to evaluate the clinical effectiveness and safety of different dose of heparin in preventing of RAO, so as to provide clinical basis for the early prevention and treatment of RAO.

## 2 Research purpose

The problem to be solved in this study is whether to use high-dose heparin is better than low-dose heparin in preventing postoperative RAO, and whether the use of high-dose heparin be used in transradial coronary angiography to effectively prevent the occurrence of RAO without increasing the risk of bleeding and other related issues.

## 3 Methods

### 3.1 Study registration

The study has been registered in Open Science Framework (OSF). Registration number is DOI 10.17605/OSF.IO/CPXJ3. Date of registration is October 11, 2020.

### 3.2 Inclusion criteria

#### 3.2.1 Type of study

A randomized controlled study (Chinese and English) comparing the effectiveness and safety of conventional dose and high-dose heparin in preventing RAO after TCA.

#### 3.2.2 Types of participants

All research subjects are in line with: patients with coronary heart disease are treated by radial coronary angiography, and unfractionated heparin is used to prevent RAO after surgery.

Inclusion criteria:

1. Patients with clinical indications of coronary angiography;
2. The study subjects clearly indicated that they were patients who underwent coronary angiography through the radial artery;
3. The intraoperative heparin dose of the included study control group was consistent with low-dose heparin (2000IU-3000IU or 50IU/kg), the experimental group meets the high-dose heparin (5000IU or 100IU/kg);
4. Allen's test negative;
5. the people with normal function of liver, kidney and blood coagulation.

#### 3.2.3 Types of Interventions

Intraoperative use of high-dose (5000IU or 100IU/kg) unfractionated heparin was injected intrathecally for preventive anticoagulation.

#### 3.2.4 Types of control groups

Intraoperative use of conventional doses (2000-3000IU or 50IU/kg) unfractionated heparin was injected intrathecally for preventive anticoagulation.

### 3.3 Exclusion criteria

1. Active bleeding or a significantly increased risk of bleeding (severe liver and kidney insufficiency, peptic ulcers, etc.);
2. History of severe peripheral vascular disease and limited radial artery access;
3. Allen's test positive;
4. with chronic wasting disease or malignant tumor;
5. allergy to contrast agents or iodine;
6. uncontrolled severe arrhythmia (ventricular arrhythmia, rapid atrial fibrillation, etc.);
7. other anticoagulants were used before and during the operation;
8. non-randomized controlled trials, including: animal experiments, reviews or case reports, etc.;
9. literature that could not effectively extract data for statistical use.

### 3.4 Outcomes

#### 3.4.1 Primary outcome measures

Radial artery occlusion: palpation of the patients radial artery followed by color Doppler ultrasonography at 1 day, 3 weeks, and 3 months after puncture. If the pulsation of the distal radial artery disappeared and the color Doppler ultrasound examination showed that the radial artery blood flow signal disappeared, it could be judged as radial artery occlusion.

#### 3.4.2 Secondary outcomes

1. Adverse events related to postoperative puncture, including skin rash, edema, local hematoma, forearm hematoma, radial artery pseudoaneurysm and compartment syndrome;
2. Incidence of gastrointestinal bleeding.

### 3.5 Data sources and search strategy

Two reviewers independently searched PubMed, EMBASE, the Cochrance Library, Web of Science, Medline, CBM Disc, CNKI, VIP to find the eligible studies. The retrieval about the randomized controlled trials of different dose of heparin in preventing the occurrence of RAO after TRA in recent years. The retrieval time is set between January 1990 and June 2020. The retrieval language is Chinese/English. We used the following terms and keywords

“heparin”, “coronary artery disease”, “percutaneous coronary intervention”, “radial artery occlusion”, etc., through the combination of the thesaurus and keywords, using Boolean logic operations to search foreign language databases. At the same time, we also reviewed the identified references for inclusion in the study, and manually retrieved potentially relevant studies to improve the recall rate of literature. Finally, it includes the related research published in English and Chinese. The retrieval strategy takes PubMed database as an example (Table 1).

**Table 1:** Searching strategy in PubMed.

Serial Number	Line
#1	(Heparin [MeSH Terms]) OR (Unfractionated Heparin [Title/Abstract])) OR (Heparin, Unfractionated [Title/Abstract]) OR (Heparinic Acid [Title/Abstract]) OR (Liquaemin [Title/Abstract]) OR (Sodium Heparin [Title/Abstract]) OR (Heparin, Sodium [Title/Abstract]) OR (Heparin Sodium [Title/Abstract]) OR (alpha-Heparin [Title/Abstract]) OR (alpha Heparin [Title/Abstract])
#2	(Coronary Artery Disease [MeSH Terms]) OR (Artery Disease, Coronary [Title/Abstract]) OR (Artery Diseases, Coronary [Title/Abstract]) OR (Coronary Artery Diseases [Title/Abstract]) OR (Disease, Coronary Artery [Title/Abstract]) OR (Diseases, Coronary Artery [Title/Abstract]) OR (Coronary Arteriosclerosis [Title/Abstract]) OR (Arterioscleroses, Coronary [Title/Abstract]) OR (Coronary Arterioscleroses [Title/Abstract]) OR (Atherosclerosis, Coronary [Title/Abstract]) OR (Atheroscleroses, Coronary [Title/Abstract]) OR (Coronary Atheroscleroses [Title/Abstract]) OR (Coronary Atherosclerosis [Title/Abstract]) OR (Arteriosclerosis, Coronary [Title/Abstract])
#3	(Percutaneous Coronary Intervention [MeSH Terms]) OR (transradial coronary angiography [Title/Abstract]) OR (Radial artery catheterization [Title/Abstract]) OR (Coronary Intervention, Percutaneous [Title/Abstract]) OR (Coronary Interventions, Percutaneous [Title/Abstract]) OR (Intervention, Percutaneous Coronary [Title/Abstract]) OR (Interventions, Percutaneous Coronary [Title/Abstract]) OR (Percutaneous Coronary Interventions [Title/Abstract]) OR (Percutaneous Coronary Revascularization [Title/Abstract]) OR (Coronary Revascularization, Percutaneous [Title/Abstract]) OR (Coronary Revascularizations, Percutaneous [Title/Abstract]) OR (Percutaneous Coronary Revascularizations [Title/Abstract]) OR (Revascularization, Percutaneous Coronary [Title/Abstract]) OR (Revascularizations, Percutaneous Coronary [Title/Abstract])
#4	(Radial Artery [MeSH Terms]) OR (radial artery occlusion [Title/Abstract]) OR (Arteries, Radial [Title/Abstract]) OR (Artery, Radial [Title/Abstract]) OR (Radial Arteries [Title/Abstract]) OR (Radial spasm [Title/Abstract]) OR (Radial occlusion [Title/Abstract])
#5	#1 AND #2 AND #3 AND #4

### 3.6 Data collection and analysis

#### 3.6.1 Selection of studies

Two researchers independently searched, managed and screened the literature through the search terms. When the 2 parties have inconsistent opinions on the inclusion or not of certain literature, the literature will be referred to the third researcher for discussion and decision; the steps of literature screening are as follows:

1. initial screening: firstly, the same literature searched in different databases is eliminated according to the title, author, journal name, abstract and other information of the article.
2. Final screening: reading and analyzing all the literature that meets the inclusion criteria, and deciding whether to include them in strict accordance with the inclusion and exclusion criteria.

**Figure 1:**
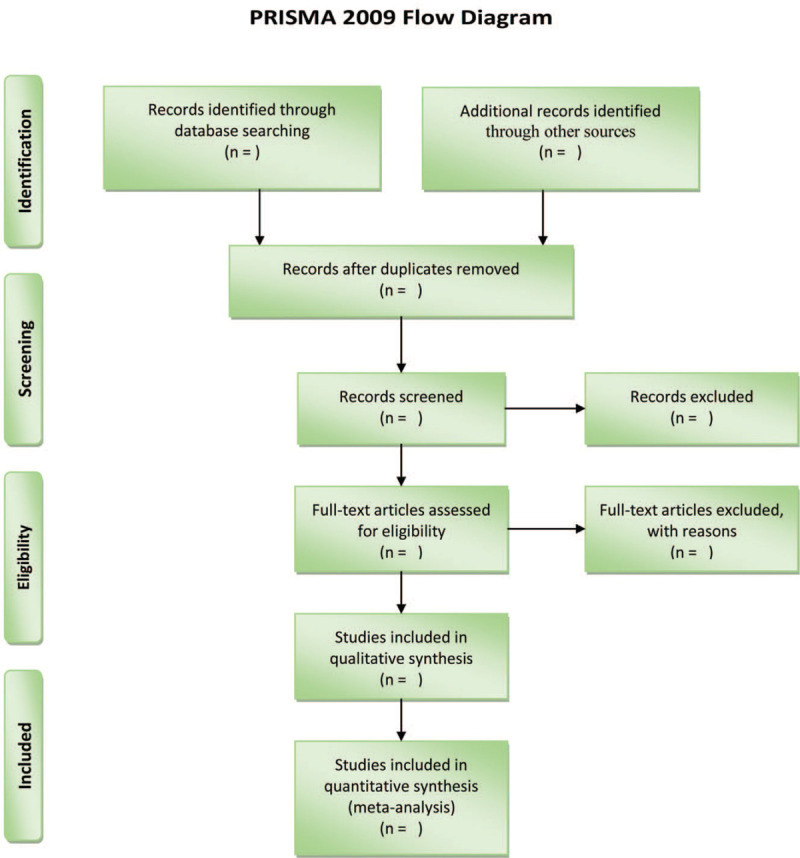
Flowchart of literature selection.

The flowchart of literature selection is shown in Figure 1.

#### 3.6.2 Data extraction and management

By using the method of Excel and manual recording, the final selected documents are extracted from the following aspects:

1. the basic information of the literature: title, author, publication years, research unit, etc.;
2. the basic data of the subjects: sample size of experimental group and control group, age, and sex of the subjects;
3. experimental intervention methods: the name, dose and course of treatment of drugs used in the experimental group and the control group;
4. the outcome indicator.

### 3.7 Assessment of risk of bias

The included studies are assessed according to the “bias risk assessment tool” recommended by Cochrane Handbook 5.0. The contents of the items are as follows:

1. whether the random sequence is generated properly;
2. whether the random assignment is hidden;
3. whether the implementer and the participants are blind (performance bias);
4. whether the outcome evaluation is blind (detecting bias);
5. whether the publication of the result data is complete;
6. whether there are selective reports;
7. whether there are other biases. For each included study, the contents of the 7 items mentioned above are described in detail, and the judgments of “low bias”, “unclear” and “high bias” are made for each item.

### 3.8 Data synthesis

The extracted data are statistically processed by using RevMan5.3 software. After inputting the data, judging the type of data and whether there is heterogeneity between the data firstly. As for the heterogeneity between data, forest map, labe chart and star chart can be used; or when *I*^2^ ≥ 50%, it indicates heterogeneity. Q-test can also be used, when *P* < .1, it indicates heterogeneity. Only when *I*^2^ < 50% and *P* > .1 in Q-test, the heterogeneity between data is low, and the fixed effect model is used to merge the effect quantity. If there is heterogeneity, using sensitivity analysis, subgroup analysis or Meta regression to find out the cause of heterogeneity. After exclusion, the heterogeneity of the remaining data can be judged again by using the method of judging heterogeneity. However, if the reason of heterogeneity can not be found, the random effect model is selected to combine the effect amount in the acceptable range of heterogeneity. The continuous variables are represented by MD or SMD and 95% CI. Inspection standard *P* = .05. When the heterogeneity test result *P* > .1 and *I*^2^ < 50%, the homogeneity is considered to be good, and the fixed effect model is used to calculate the combined effect amount; when the heterogeneity test result *P* ≤ .1 or *I*^2^ ≥ 50%, the heterogeneity is considered to be significant, and the random effect model is used to calculate the combined effect amount. Finally, a meta-analysis of combined effect amount is performed. For count data or ordered data, RR or OR can be used for quantitative comparison. And the 95% confidence interval is calculated. Subsequently, the sensitivity analysis of the results is carried out by replacing the effect model and excluding a certain study. Afterwards, perform a meta-analysis to merge effect size. Finally, the “funnel chart” method is used to analyze potential publication bias. It also explains and analyzes the combined effect amount of the meta-analysis.

#### 3.8.1 Sensitivity analysis

If the heterogeneity is large between the studies, sensitivity analysis is needed. After deleting the included studies one by one, a new meta-analysis is carried out to compare that whether the effect quantity and heterogeneity change before and after deletion. If the heterogeneity changes after deleting a certain study, and the amount of effect is still statistically significant, then it is considered that there is heterogeneity in the study, so it is necessary to further compare and analyze the source of heterogeneity. If the after effect value and heterogeneity change of each study are not obvious, then the results are stable and reliable.

#### 3.8.2 Publication bias

The publication bias is shown by inverted funnel chart. The ordinate is the sample size of the study, or the standard error or accuracy (the reciprocal of the standard error). As the sample size is smaller, the distribution is more dispersed; the larger the sample size, the more concentrated the distribution, and the more points in the funnel graph are concentrated in the top area.^[17]^ If there is no bias, the chart is symmetrical and funnel-shaped. On the contrary, if the chart is asymmetry and biased, and it indicates that there is publication bias.

#### 3.8.3 Ethics and dissemination

Ethics and dissemination are not applicable to this study.

## 4 Discussion

At present, the number of patients who have completed percutaneous coronary intervention has increased year by year. Because of its advantages such as more comfort and safety, shorter hospital stay and higher patient compliance, transradial coronary angiography has been selected by more clinicians and patients. At present, more than 90% of percutaneous coronary intervention in China is completed through radial artery approach. But at the same time, there are many special complications of radial artery approach (such as radial artery occlusion, forearm hematoma, radial artery pseudoaneurysm, osteofascial compartment syndrome, etc.), of which radial artery occlusion is the most common, especially in elderly and female patients. The mechanism of RAO involves many different theories. At present, the most convincing mechanism is the occurrence of local thrombosis and radial artery spasm. Radial artery thrombosis mainly occurs in the process of TCA due to radial artery puncture and guide wire insertion, improper operation when inserting a catheter, weak manipulation, or blood vessel itself, which leads to vascular intima injury, local exposure of collagen fibers and tissue factors, and activation of internal, exogenous blood coagulation pathway, leading to local platelet and fibrin aggregation, forming thrombus at the location of vascular injury, and ultimately leading to the occurrence of RAO.^[18]^ Radial artery spasm mainly occurs on the basis of vascular related pathological changes, such as diabetic peripheral vascular disease, arteritis, atherosclerosis. During the invasive operation, vascular smooth muscle contracts spontaneously after receiving friction stimulation, resulting in radial artery hemodynamic disorder. In contrast, the mechanism of thrombosis is more closely related to the occurrence of RAO, and anticoagulation is the main means to prevent RAO.^[19]^ There are many methods of preventive anticoagulation. As a classical anticoagulant for effective prevention of thrombosis, unfractionated heparin can be combined with antithrombin to turn it into a rapid thrombin inhibitor, catalyzing the inactivation of coagulation factors IIa, IXa, Xa, Xia, and XIIa, so as to play an anticoagulant role.^[20]^ Unlike low-molecular-weight heparin, unfractionated heparin can be neutralized by protamine, which can effectively prevent bleeding and other complications caused by excessive anticoagulation to a certain extent. Currently, unfractionated heparin has been widely used in related operations through the radial artery. There is no unified opinion on the amount of heparin before TCA operation at home and abroad. Therefore, this study searches recent randomized controlled trials in preventing the RAO after TCA with different dose of heparin, and uses a systematic review to evaluate the effects of different dose of heparin in preventing the RAO. The clinical effectiveness and safety of this study will provide a theoretical basis for the early prevention and treatment of RAO.

## Author contributions

**Conceptualization:** Ling Zhao, Fengde Li.

**Data curation:** Ling Zhao, Huijing Zhang.

**Formal analysis:** Yanlei Pang, Yong Li.

**Funding acquisition:** Fengde Li.

**Investigation:** Ling Zhao, Yanlei Pang, Qun Zheng.

**Methodology:** Ling Zhao, Yanlei Pang, Qun Zheng.

**Resources:** Yanlei Pang, Fengde Li.

**Software:** Ling Zhao, Yanlei Pang, Huijing Zhang.

**Supervision:** Fengde Li.

**Writing – original draft:** Ling Zhao, Yanlei Pang, Huijing Zhang, Yong Li, Qun Zheng.

**Writing – review & editing:** Fengde Li.
